# SHAPE Selection (SHAPES) enrich for RNA structure signal in SHAPE sequencing-based probing data

**DOI:** 10.1261/rna.047068.114

**Published:** 2015-05

**Authors:** Line Dahl Poulsen, Lukasz Jan Kielpinski, Sofie R. Salama, Anders Krogh, Jeppe Vinther

**Affiliations:** 1 Department of Biology, University of Copenhagen, DK-2200 Copenhagen N, Denmark; 2 Center for Biomolecular Science and Engineering, and Howard Hughes Medical Institute, University of California Santa Cruz, Santa Cruz, California 95064, USA

**Keywords:** RNA structure, SHAPE, Selection, NPIA, sequencing

## Abstract

Selective 2′ Hydroxyl Acylation analyzed by Primer Extension (SHAPE) is an accurate method for probing of RNA secondary structure. In existing SHAPE methods, the SHAPE probing signal is normalized to a no-reagent control to correct for the background caused by premature termination of the reverse transcriptase. Here, we introduce a SHAPE Selection (SHAPES) reagent, N-propanone isatoic anhydride (NPIA), which retains the ability of SHAPE reagents to accurately probe RNA structure, but also allows covalent coupling between the SHAPES reagent and a biotin molecule. We demonstrate that SHAPES-based selection of cDNA–RNA hybrids on streptavidin beads effectively removes the large majority of background signal present in SHAPE probing data and that sequencing-based SHAPES data contain the same amount of RNA structure data as regular sequencing-based SHAPE data obtained through normalization to a no-reagent control. Moreover, the selection efficiently enriches for probed RNAs, suggesting that the SHAPES strategy will be useful for applications with high-background and low-probing signal such as in vivo RNA structure probing.

## INTRODUCTION

Under physiological conditions, RNA has the ability to form structures through internal base-pairing. This additional layer of information encoded in the RNA sequence will in many cases be key to understanding the function of RNA molecules. This is true for the abundant noncoding RNAs, but also for protein-coding mRNAs, which often contain functional regulatory RNA structures. For highly conserved structural RNAs, comparative data allow the secondary structure to be deduced (Gutell et al. 2002). In the absence of conservation, computational methods based on minimization of free energy or stochastic context-free grammars can be used to confidently predict the secondary structure (Washietl et al. 2012). For many RNAs, however, computational identification of the biologically relevant secondary structure remains challenging. A successful approach to improve RNA secondary structure prediction has been to use experimental probing data to guide the computational predictions. In particular, it has been shown that data from Selective 2′-Hydroxyl Acylation analyzed by Primer Extension (SHAPE) experiments significantly improve secondary RNA structure prediction (Deigan et al. 2009; Weeks and Mauger 2011). SHAPE reagents react with the 2′ OH group of the ribose present in each nucleoside of an RNA in a manner that is largely independent of base identity (Wilkinson et al. 2009), but depends on the ribose adopting specific conformations that are sampled by flexible regions of the RNA, but not by base-paired regions (Merino et al. 2005; McGinnis et al. 2012). After SHAPE probing, primer extension by reverse transcriptase will result in the synthesis of cDNAs that terminate at positions immediately before SHAPE adducts. Termination will also occur at positions of RNA degradation, modifications or other features that may cause spontaneous termination of reverse transcription, such as stable RNA structures (Harrison et al. 1998). Thus, the SHAPE probing signal will be mixed with a background signal, which will confound RNA structure prediction. The signal to background ratio obtained in a particular SHAPE probing experiment depends on the efficiency of probing and the subsequent reverse transcription based detection. For SHAPE probing reactions based on a single location for hybridization of reverse transcription primers, a control reaction without probing reagent is typically performed in parallel with the probed reaction. The control data can be used to normalize the data from the probed reaction to give estimates of the SHAPE reactivity of each position. Robust methods for performing the normalization and dealing with the decay of signal, which occurs as a result of reverse transcriptase termination, have been developed. These methods rely either on the estimation of parameters for the background in a sophisticated probabilistic model (Aviran et al. 2011) or an α parameter, which denotes a scaling factor for determining the level of background present in the probed data (Karabiber et al. 2013). However, these normalization methods require a control sample, which accurately reflects the background found in the probed sample, have so far only been implemented for single primer experiments and are influenced by the signal to background ratio in the samples.

During the last couple of years, the throughput of RNA structure-probing methods has increased significantly by adaptation of the methods to massively parallel sequencing (Kertesz et al. 2010; Underwood et al. 2010; Lucks et al. 2011; Seetin et al. 2014). For SHAPE probing, it has been demonstrated that it is possible to probe many RNAs in parallel using a primer that hybridize to one specific position in the 3′ end of the RNA (Lucks et al. 2011), but so far SHAPE probing has not been adapted to probing of long RNAs. In contrast, methods based on selective cleavage of single and double stranded regions by enzymatic means have been successfully applied to complex mixtures of long RNA molecules (Kertesz et al. 2010; Underwood et al. 2010). Using these methods, the secondary structure of thousands of RNAs were probed in parallel to provide the first global view of RNA structure (Kertesz et al. 2010; Underwood et al. 2010) and more recently to evaluate the effects of SNPs on RNA structure through probing of the transcriptomes of related humans (Wan et al. 2014). However, enzymatic methods are limited to conditions that support enzymatic activity, whereas chemical probing of RNA structure can be applied in many different conditions, including the intracellular environment. This was recently demonstrated for *Arabidopsis thaliana* seedlings (Ding et al. 2014) and yeast, mouse, and human cells (Rouskin et al. 2014) using dimethylsulfate (DMS), which efficiently penetrates cell membranes and methylate unprotected adenines and cytosines. Detection of reverse transcriptase terminations by next-generation sequencing allowed the first global views of in vivo RNA structure. The continued application and improvement of such in vivo methods is central for identification and characterization of functional RNA structures. An advantage of SHAPE probing compared with DMS probing is that it provides information for all positions rather than only the adenine and cytosine positions and for in vitro RNA structure-probing experiments SHAPE has been the preferred method. The development of new SHAPE reagents based on imidazolide chemistry, which can probe RNA structure inside living cells (Spitale et al. 2013), and the adaption of well-known SHAPE reagents to in vivo probing (Tyrrell et al. 2013) suggest that SHAPE methodology eventually can be applied to global probing of in vivo RNA structure. However, it is clear that global in vivo SHAPE probing will be more challenging than the probing of single RNA molecules in vitro. First, in vivo SHAPE RNA structure probing requires the SHAPE reagent to cross cell membranes and will encounter the entire repertoire of RNA molecules present in the target cell population. This makes probing events for a given RNA of interest less frequent than what is typically found with in vitro experiments. Second, global probing requires priming of reverse transcription in many different locations, which makes normalization more difficult and potentially increases the background. Combined, these effects can make it difficult to distinguish the signal originating from in vivo structures from that of background noise.

Here, we describe a novel SHAPE Selection (SHAPES) reagent, *N*-propanone isatoic anhydride (NPIA), which retains the ability of SHAPE reagents to accurately probe RNA structure, but also allows covalent coupling to a biotin molecule. We demonstrate that RNase I treatment of cDNA/RNA–NPIA–biotin hybrids followed by selection on streptavidin beads enriched for probed RNAs and effectively removes the large majority of background signal present in SHAPE probing data. Moreover, we have adapted SHAPES to randomly primed reverse transcription and sequencing-based readout of reverse transcription termination sites, making the probing of long RNAs without normalization with a no-reagent control possible. We believe that the SHAPES strategy will provide an alternative to regular SHAPE methods for in vitro probing of RNA structure and will be especially useful for applications with high-background and low-probing signal such as in vivo structure probing.

## RESULTS

### NPIA reacts with RNA and can be coupled to biotin

**FIGURE 1.**
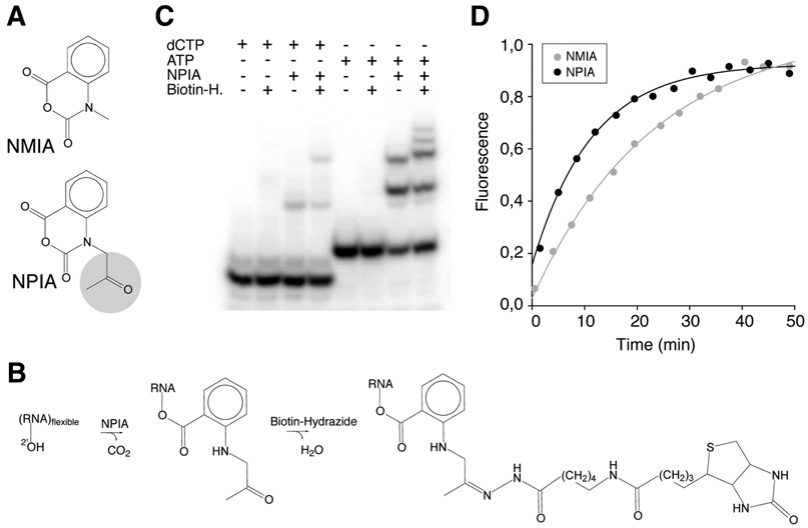
SHAPE Selection chemistry. (*A*) Chemical structures of *N*-methyl isatoic anhydride (NMIA) and *N*-propanone isatoic anhydride (NPIA). The *N*-methyl group of NMIA is exchanged to an *N*-propanone group in NPIA (marked in gray). (*B*) Reaction of NPIA with RNA and biotin (long arm) hydrazide. RNA in a flexible conformation is acylated by NPIA via the 2′-OH group, forming a stable 2′-O-adduct containing an *N*-propanone group. The *N*-propanone group is then biotinylated with biotin (long arm) hydrazide. (*C*) Polyacrylamide gel electrophoresis with products obtained by reacting radioactively labeled dCTP and ATP with NPIA and subsequently with biotin (long arm) hydrazide (Biotin-H). dCTP and ATP have reduced mobility in the polyacrylamide gel after reaction with NPIA, and the migration is further reduced upon reaction with biotin (long arm) hydrazide. (*D*) Comparative hydrolysis reactivity of NMIA (gray) and NPIA (black). The reaction with water was measured as an increase in fluorescence over time at 25°C, and the lines represent nonlinear regression to all data points.

Inspired by the efficient selection methods for identification of transcription start sites by cap analysis of gene expression (CAGE) (Shiraki et al. 2003; Takahashi et al. 2012), we set out to develop a novel SHAPE method based on selection. In CAGE the 5′ cap is biotinylated through oxidation of the ribose diol to aldehyde groups, which are then reacted with biotin–hydrazide. We obtained *N*-propanone isatoic anhydride (NPIA), which is identical to the canonical SHAPE reagent *N*-methyl isatoic anhydride (NMIA), except that the *N*-methyl group has been exchanged with an N-propanone group (Fig. 1A). Like aldehydes, ketones react specifically with hydrazides and we therefore hypothesized that the NPIA reagent would allow a biotin molecule to be coupled specifically to SHAPE probed positions (Fig. 1B). We tested the reactivity of NPIA toward the ribose hydroxyl groups by reacting it with radioactive dCTP and ATP. As expected for a SHAPE reagent, we observe a single shift of the dCTP as a result of acylation of the 3′ hydroxyl group and a double shift of the ATP, through single and double acylation of the two hydroxyl groups (Fig. 1C). Subsequent reactions with biotin–hydrazide leads to a further shift of the NPIA–dCTP adduct and the NPIA–ATP adducts, but not dCTP or ATP alone. For NPIA reacted ATP the reaction with biotin–hydrazide leads to a shift of the mono-NPIA–ATP adduct to a position just above the double acylated ATP, consistent with the biotin having a larger MW than the NPIA molecule. In addition the double acylated ATP is shifted and generates two upper bands corresponding to the single and double biotinylated forms. The presence of the shifts and the efficient shift of the double acylated ATP adduct indicates that the coupling between the propanone group and the biotin–hydrazide is relatively efficient. The 2′ acylation of RNA by SHAPE reagents competes with the hydrolysis of the SHAPE reagents. For isatoic anhydride-based SHAPE reagents hydrolysis creates fluorescent 2-amino-benzoates, allowing the rate of hydrolysis to be monitored (Merino et al. 2005). We find that NPIA has a half-life of 5.9 min, which is shorter than the canonical SHAPE reagent NMIA, but not as short as the widely used reagent 1M7 (Fig. 1D; Mortimer and Weeks 2007).

### SHAPES probing of the *Bacillus subtilis* RNase P RNA

**FIGURE 2.**
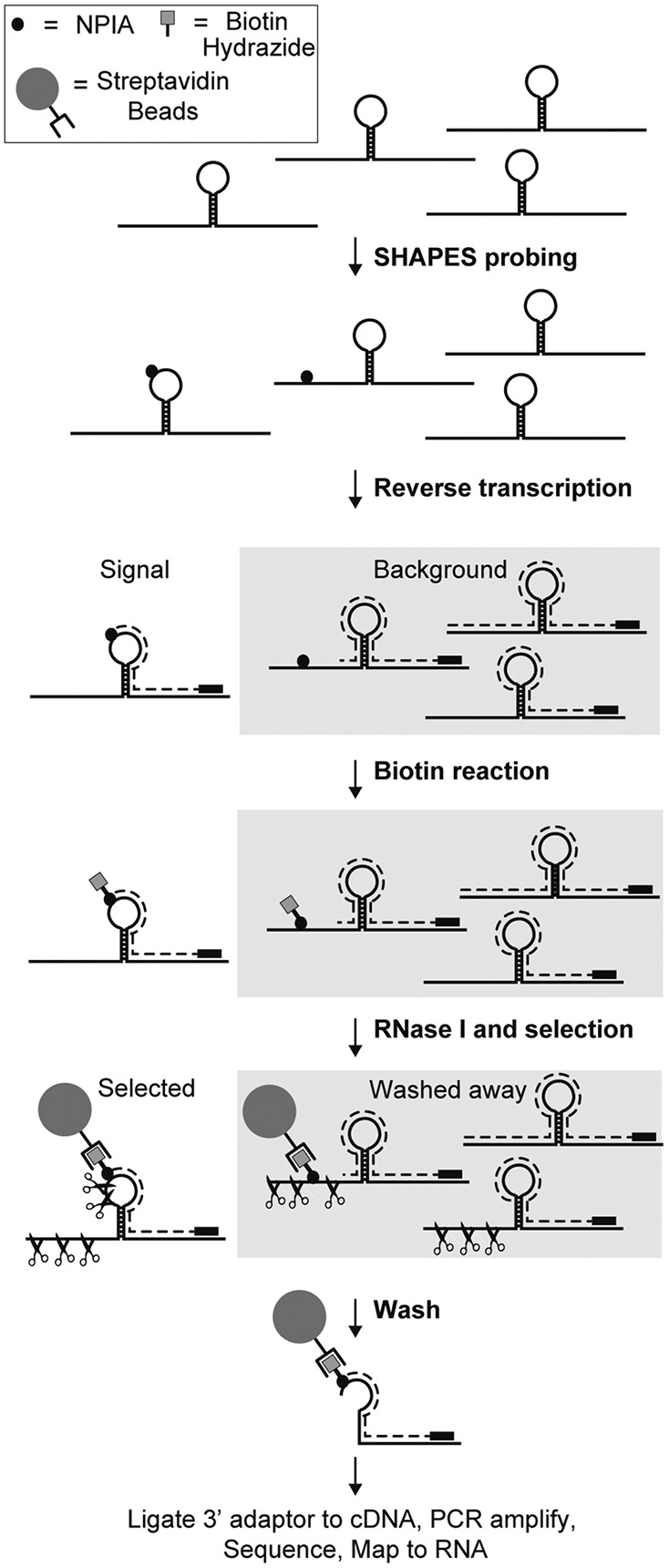
SHAPES strategy. Schematic representation of the SHAPES strategy. RNA is probed with NPIA and reverse transcription primers are extended. Some of the produced cDNAs will terminate prematurely or at the very 5′ end of the RNA causing background, whereas others will reach the NPIA modification to give cDNAs that contain the structural information. The propanone group of NPIA allows biotin–hydrazide to be coupled to the reagent and subsequent treatment with RNase I will cleave all single stranded RNA. Selection on streptavidin beads will wash away cDNAs caused by premature termination or 5′ end run-off, leaving the cDNA that contain the structural information.

**FIGURE 3.**
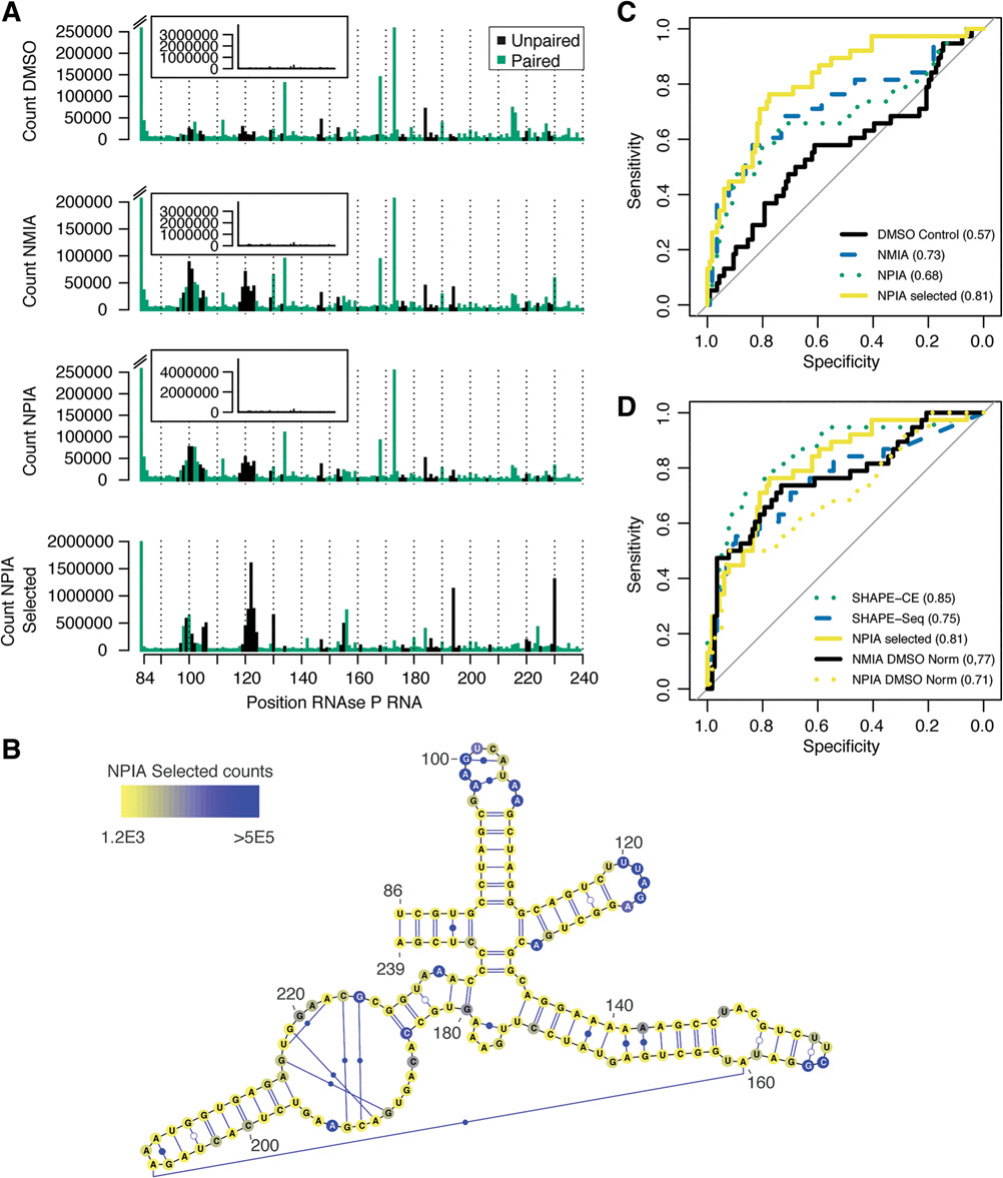
SHAPES probing of *RNase P* specificity domain RNA. (*A*) cDNA termination counts for probing of the *RNase P* specificity domain RNA from the DMSO control sample, NMIA probed sample, NPIA probed sample, and the NPIA probed and selected sample. The coloring of the bars corresponds to the base pair (including noncanonical) annotation of the RNase P RNA as observed in the crystal structure (Krasilnikov et al. 2003), with black being unpaired and green being base-paired. The *insets* show the plot including the 5′ run-off position for comparison with the selected NPIA sample, where this position is included in the main plot. (*B*) The base-pairing (both canonical and noncanonical) of the RNase P specificity domain RNA observed in the crystal structure is shown in the figure. Individual positions are colored by their termination count observed in the selected NPIA sample. (*C*) Receiver-operating characteristic (ROC) curves for the termination counts obtained from the different samples shown in *A* using the base-pairing information shown in *B* as the binary classifier. The area *under* the ROC curve (AUC) for the different samples is indicated in the legend. (*D*) ROC curves based on the base-pairing information shown in *B* for the SHAPE reactivities obtained using capillary electrophoresis and DMSO control normalization (SHAPE-CE) (Mortimer and Weeks 2007), SHAPE reactivities obtained using sequencing and DMSO normalization (SHAPE-Seq) (Lucks et al. 2011), the NPIA-selected count obtained in this study, and finally the NMIA and NPIA data obtained in this study normalized with the DMSO data obtained in this study. The area *under* the ROC curve (AUC) for the different samples is shown in the legend.

The ability of NPIA to react with RNA and be coupled to biotin makes it possible to perform a CAGE-like selection strategy on SHAPE probed RNA (Fig. 2). The selection procedure enriches for cDNAs that terminate specifically at probed positions, while RNAs that have not been probed or RNAs for which the reverse transcription is prematurely terminated will be washed away during the selection. As a first proof of the SHAPES concept, we probed the specificity domain of the *B. subtilis RNase P* RNA with NMIA, NPIA and with DMSO as a no-reagent control. Following the SHAPE-Seq set-up (Lucks et al. 2011) we primed the RNA with a single specific reverse transcription primer containing an Illumina adaptor 5′ overhang. After extension of the reverse transcription primer, some samples were subjected to SHAPES selection as outlined in Figure 2. Subsequently, adaptors were ligated to the 3′ ends of cDNAs using CircLigase enzyme and libraries were synthesized by PCR amplification using indexed primers. After size selection to remove reverse transcription primer–adaptor products, the libraries were sequenced using the Illumina single read protocol. The resulting reads were mapped to the RNase P RNA and termination events were counted (Fig. 3A). We find that the termination counts from NPIA and NMIA unselected probing reactions are strongly correlated (*R* = 0.98), indicating that NPIA and NMIA react with RNA in a nearly identical fashion. Termination counts from the two reactions are also quite strongly correlated to the termination counts from the DMSO control (*R* = 0.88 for both), demonstrating that both probing reactions contain a substantial amount of background. Reactions were carried out at conditions that favored single-hit kinetics and as expected all three reactions have a high percentage of reads mapping to the RNase P 5′ end (Fig. 3A, insets). After selection of the samples according to the SHAPES scheme (Fig. 2), the fraction of reads mapping to the 5′ end of the RNA remains essentially unchanged for the selected NMIA sample (0.66 versus 0.64) and the termination count profile for the selected NMIA sample still correlates strongly with the profile from the DMSO sample (*R* = 0.83) (data not shown). In contrast, for the selected NPIA experiment the correlation with the control sample is much lower (*R* = 0.63) and the run-off count constitutes a much smaller fraction of the total count in the selected sample compared with the unselected sample (0.10 versus 0.71). Moreover, the dominant termination count peaks observed in the DMSO sample are not present, demonstrating that the signal caused by nonprobed RNAs or premature termination of reverse transcriptases has been effectively depleted (Fig. 3A). To quantify the amount of structural signal present in the different samples, the data can be plotted as receiver-operating characteristic (ROC) curves with the base-paired/unpaired information for RNase P RNA as the binary classifier and the area under the ROC curve (AUC) can be determined. The crystal structure of the RNase P specificity domain shows that this RNA is compactly folded and contains many noncanonical base-pairing interactions (Krasilnikov et al. 2003). For our analysis, we used all the base-pairing interactions that were observed in the crystal structure (Fig. 3B). As expected, the data from the DMSO sample contain little structural information and probing with either NMIA or NPIA increases the amount of structural information present in the data (Fig. 3C). Importantly, compared with the unselected NPIA sample, we find that the selection procedure increases the structural information present, demonstrating that SHAPES efficiently enriches for the reverse transcription termination events that are caused by probing reagent. Next, we compared the structural signal present in the selected NPIA sample with the SHAPE structure signal previously obtained for the *RNase P* RNA either by capillary electrophoresis (SHAPE-CE) (Mortimer and Weeks 2007) or sequencing (SHAPE-Seq) (Fig. 3D; Lucks et al. 2011). Both of these data sets were obtained by the calculation of SHAPE reactivities using a DMSO negative control. We find that our nonnormalized NPIA-selected sample contains more structural information than that present in the data from the SHAPE-Seq study (Lucks et al. 2011), whereas both the sequencing-based studies contain less signal than what was obtained using capillary electrophoresis as readout (SHAPE-CE) (Mortimer and Weeks 2007). For comparison, we also performed normalization of our NMIA and NPIA samples using a DMSO normalization strategy very similar to the one developed by Weeks and colleagues (Karabiber et al. 2013). The structure signal obtained with our method is similar to the signal obtained with SHAPE-Seq (Fig. 3D; Lucks et al. 2011). Our structure signal is most convincing for the 5′ part of the RNA, possibly because of size selection issues in the library preparation, which biases against shorter sequencing fragments and the many noncanonical base pair interactions present in the internal loop positions185–196 and 217–225 (Fig. 3B).

### SHAPES probing of the *Escherichia coli* 16S rRNA

**FIGURE 4.**
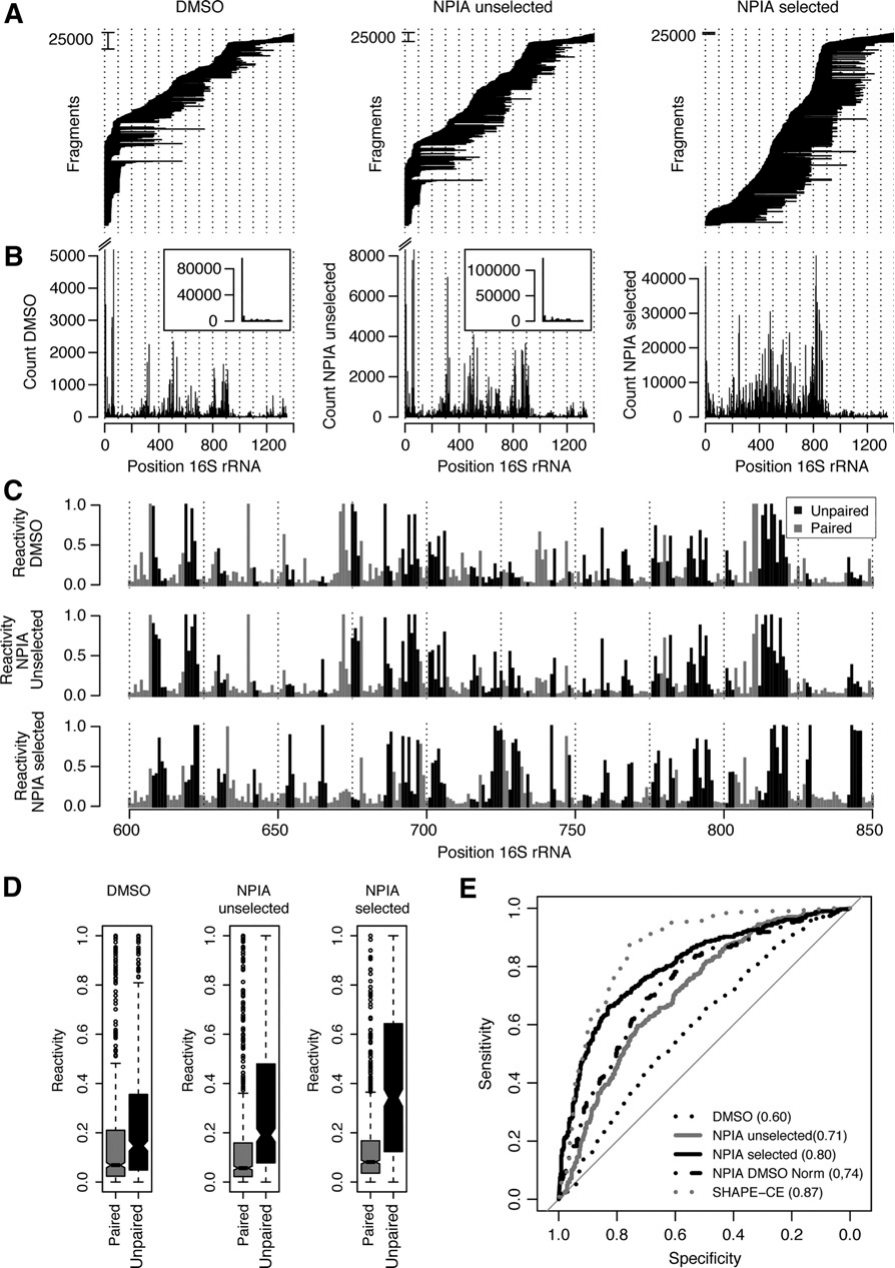
SHAPES probing of 16S rRNA. The figure shows data for the *E. coli* 16S rRNA obtained from three samples: (*left*) the probing procedure without any probing reagent added, (*center*) probing with NPIA, and (*right*) probing with NPIA plus subsequent selection. (*A*) Plot of the fragments obtained for the different samples. *Left* ends of the lines show the position corresponding to the cDNA termination position and the *right* ends the position corresponding to cDNA priming. The height of the bar to the *left* of the plot indicates how many fragments were identified in the different sequencing samples. (*B*) The termination count for the different samples. For DMSO control and the NPIA unselected samples the bar showing the count for the 5′ run-off position is cut in the main plot, but shown in the *inset* for comparison with the selected NPIA sample. (*C*) SHAPE reactivities for 16S rRNA region 600–850 for the DMSO, NPIA-unselected, and NPIA-selected samples. The shading of the bars shows the secondary structure annotation of the 16S rRNA with black being unpaired and gray being paired. (*D*) Boxplots showing the distributions of SHAPE reactivities for the DMSO, NPIA-unselected, and NPIA-selected samples stratified by the secondary structure annotation. (*E*) ROC curves for the DMSO, NPIA-unselected, NPIA-selected samples, and SHAPE data generated using capillary electrophoresis and DMSO control normalization (SHAPE-CE) (Deigan et al. 2009) using the secondary structure annotation of the 16S rRNA as the binary classifier. The area *under* the ROC curve (AUC) for the different samples is shown in the legend.

Next, we wanted to further validate the selection procedure and to extend SHAPES to the probing of long RNAs by using random priming in the reverse transcription. We purified *E. coli* ribosomes using a gentle purification protocol to preserve the overall RNA fold (Deigan et al. 2009). The resulting RNA was used for a control DMSO reaction or probed with NPIA with or without selection. The processed samples were sequenced with the Illumina paired-end protocol to provide information of the position of priming (right end of fragment) and reverse transcription termination (left end of fragment) (Fig. 4A). SHAPES termination counts can be obtained by summing termination events for each position (Fig. 4B). Again, it is clear that the selection procedure significantly reduces the number of fragments terminating at the 5′ end of the RNA, resulting in more termination counts in the body of the RNA and presumably a reduction of signal from reverse transcription pretermination (compare Fig. 4B insets with the termination count for the NPIA-selected sample). To further investigate selection efficiency, we spiked in vitro transcribed *RNase P* RNA and β-actin mRNA into the *E. coli* RNA before and after performing NPIA probing, respectively. Thus, the RNase P RNA should be probed and selected during our SHAPES procedure, whereas the β-actin mRNA should not be probed and therefore not selected. We find that the ratio between the count of mapped fragments for β-actin RNA and the *RNase P* RNA is 3.2 for the unselected NPIA sample, whereas the NPIA-selected sample has a ratio of 0.39, again supporting that our selection works efficiently. Surprisingly, we find that the random priming during reverse transcription is quite biased, which causes some regions to have higher coverage than others. We do not expect this to affect experiments focused at finding relative differences between two samples, but it is problematic for obtaining a structural signal for RNA structure prediction. In most cases neighboring positions will experience similar coverage and we therefore used a 70-nt sliding window approach to normalize all positions by the count from the 95 percentile in the window. Positions with a count higher than the 95 percentile were set to a SHAPES reactivity of one and for each position the SHAPES reactivity was calculated as the average of the SHAPES reactivities obtained in each of the overlapping windows (Fig. 4C). A window size of 70 is a compromise between the need for capturing the local information and having enough positions in the window to get an accurate estimation of the 95 percentile, but similar results can be obtained with window sizes in the range between 30 and several hundreds. In agreement with the results obtained for the RNase P molecule, we find that selection removes many of the dominant peaks observed both in the DMSO control sample and the NPIA unselected sample. Moreover, when the reactivities for the samples are stratified by the base-pairing information of each position based on the phylogenetic structure annotation of the 16S rRNA (Cannone et al. 2002), it is clear that NPIA probing shifts SHAPES reactivities of unpaired positions toward higher reactivity values, which is what would be expected for a functional SHAPE reagent (Fig. 4D). After introduction of the selection step, most positions with high SHAPES reactivity map to loops and bulges of the secondary structure of the 16S rRNA (Supplemental Fig. 1) and the distribution of reactivities for unpaired positions is further shifted toward higher values (Fig. 4D). To more formally quantify the differences in structure signal between the different samples, we calculated AUC for the DMSO, NPIA and NPIA-selected samples using the 16S rRNA secondary structure phylogenetic annotation as the binary classifier. A weak structure signal is present in the data from the DMSO sample (AUC = 0.60) and as expected NPIA probing of the RNA increases the structure signal present (AUC = 0.71) (Fig. 4E). SHAPES selection leads to a further improvement in the amount of structure signal obtained and provides a robust structure signal (AUC = 0.80) (Fig. 4E). As observed for the RNase P RNA, the SHAPES reactivities contain less structure signal than provided by a DMSO normalized SHAPE experiment detected by capillary electrophoresis (Fig. 4E; Deigan et al. 2009). However, when we normalize our unselected NPIA data with the data obtained from the DMSO control using a normalization strategy similar to the one previously described by Weeks and coworkers (Karabiber et al. 2013), we find that our DMSO normalized SHAPE data contain less structure signal than we observe with the SHAPES method (Fig. 4E).

## DISCUSSION

Here, we describe SHAPE Selection (SHAPES), which is a novel strategy for probing of secondary RNA structure. We demonstrate that SHAPES effectively removes the background signal present in SHAPE-based RNA structure-probing data by selection of informative cDNA–RNA hybrids from cDNAs caused by pretermination of reverse transcriptase or RNA 5′ end run off. Our method is conceptually similar to the CAGE method for identifying the capped end of mRNAs, which has been extensively validated (Shiraki et al. 2003; Takahashi et al. 2012). The major difference between the two methods is that the biotin–hydrazide reagent in CAGE reacts with the oxidized 5′ cap of mRNAs and in SHAPES with the ketone group on the NPIA reagent. For the SHAPE selection, we use the washing conditions that has been optimized for CAGE (Takahashi et al. 2012), including RNase I digestion of the RNA–cDNA hybrids combined with a 65°C denaturation step. This prevents the capture of multiple cDNAs hybridized to the same RNA molecule, because the long random primers used in the protocol in most cases will prime with mismatches, allowing RNase I to cleave at the priming positions. In addition this denaturation step ensures that cDNAs terminated prematurely at non-SHAPES modified positions cannot be selected through involvement in secondary structures that contain SHAPES modifications elsewhere.

SHAPES offers two major advantages compared with standard SHAPE. First, the selection procedure makes the no-reagent control typically used for SHAPE probing experiments unnecessary, meaning that only one sample needs to be sequenced. This will reduce the costs associated with this kind of sequencing-based probing experiment. On the other hand, the selection step increases the time required to perform the experiment with an additional day. We believe that in this type of experiment, where data analysis is a major part of the project, most researchers would like to get the double amount of data for the same cost at the expense of spending an additional day in the laboratory. Second, in cases with high background, the selection will enrich for the probed RNAs, thereby allowing RNA structure-probing data to be obtained. For the recently published DMS based in vivo RNA structure probing in human cells, stringent size selection was performed first on the probed and fragmented RNA and subsequently on the cDNA obtained by reverse transcription (Rouskin et al. 2014). Through this double-size selection procedure, the background signal in the data is reduced and the cDNAs that terminated on the DMS modifications are enriched compared with the nonprobed RNA fragments (Rouskin et al. 2014). The SHAPES strategy also results in reduced background by removing not only the nonprobed RNA, but also cDNA that are terminated before the probing position, suggesting that SHAPES strategy will be useful for in vivo RNA structure probing and an important alternative to the published DMS methods (Ding et al. 2014; Rouskin et al. 2014). In this study, we have focused on obtaining SHAPES reactivity values that directly reflect the local nucleotide flexibility of the RNA strand. As demonstrated by regular SHAPE probing experiments such data can be used for validating secondary RNA structure models or to guide computational methods for predicting secondary RNA structure (Deigan et al. 2009). In other cases, the aim is to identify RNA regions that undergo structural rearrangements and we expect SHAPES data to be suitable for this kind of relative comparison between samples.

In our 16S rRNA experiment, we have introduced random priming for sequencing-based SHAPE structure probing, which facilitates the structural probing of long RNA molecules. Our priming strategy is similar to the strategy used for priming in the DMS-based Structure-Seq (Ding et al. 2014) and in HRF-Seq (Kielpinski and Vinther 2014). We find that the priming is quite biased, but still sufficiently spread out over the RNA to provide a good probing signal across the RNA. In these experiments, we have used a random primer ending in either G or C to improve efficiency of reverse transcription, which may have contributed to the biased priming that we observe. In future studies, we plan to use a truly random primer.

In this study, we demonstrate that NPIA can be used as a SHAPES reagent, allowing both structure probing and coupling to a solid support. We have tested the NPIA reagent for in vivo use, but found that the reactive propanone group reacts with other molecules than RNA in vivo. Thus to use the SHAPES strategy inside cells, we have synthesized SHAPES probing reagents that have the biotin molecule coupled directly to the SHAPE reactive group and pilot experiments show that these molecules can enter human cells and probe RNA structure. In this way, the probed RNA is directly biotin labeled and can be used directly for selection. Alternatively, other chemical methods for coupling to a solid support, such as click chemistry, could be used to allow in vivo SHAPES.

Our SHAPES strategy produces a robust RNA structure signal both for the *B. subtilis RNase P* specificity domain RNA and for the *E. coli* 16S rRNA. However, for both RNAs our signal is not as strong as the signal obtained by the Weeks group using the traditional SHAPE setup and normalization with a DMSO control (Mortimer and Weeks 2007; Deigan et al. 2009). On the other hand, the raw NPIA-selected termination counts for the RNase P RNA obtained by our method contains a structure signal that is on par with the signal obtained with the DMSO control normalized SHAPE-Seq method (Fig. 3C; Lucks et al. 2011) and for the 16S rRNA, we find that our SHAPES data contain more structure signal than the unselected NPIA data normalized with the DMSO control (Fig. 4E). This shows that current methods for sequencing-based readout of SHAPE probing remains inferior to capillary-based readout, except with respect to the throughput. A likely explanation for the lower amount of structure signal obtained in sequencing-based methods is the biases introduced by using sequencing as readout for the structure signal. In particular, ligation to the cDNA 3′ end, PCR amplification and size selection of the library may cause bias compared with data produced by capillary electrophoresis. However, we expect that the quality of the sequencing-based SHAPES data will be improved in the future. First, to reduce the effect of PCR bias it is possible to introduce random barcodes in the ligation adaptor, thereby allowing identical fragments produced by PCR amplification to be identified and removed (Kielpinski and Vinther 2014). Second, our sequencing libraries are size-selected to remove a PCR product resulting from ligation of the reverse transcription primer to the 5′ adaptor and both bead based purification during library preparation and Illumina sequencing potentially further increase the size bias. An advantage of the stringent SHAPES selection is the efficient removal of the reverse transcription primer. This reduces the amount of reverse transcription primer–adaptor artifacts and is the primary reason that we have more fragments that map to the 16S rRNA for the selected NPIA sample compared with the unselected NPIA sample and the DMSO sample (Fig. 4). In future experiments that probe complex pools of RNAs, it should be possible to estimate the global fragment size distribution and an average drop-off rate of reverse transcriptase, which in combination would make it possible to correct for the effects of size selection. Third, we find that the ligation of the adaptor to the 3′ end of cDNA by CircLigase is biased with cDNAs ending in T and A ligating more efficiently than those ending with G and C. In our experience, the ligation bias is reproducible, indicating that a control with randomly fragmented complex RNA could be used for estimating the ligation bias, which in turn could be used for correction to improve data from sequencing-based SHAPE probing experiments.

In conclusion, we demonstrate that SHAPES removes the background caused by premature termination of reverse transcriptase and 5′ end run-off from SHAPE-based probing data. In addition, we have adapted the SHAPES method to allow random priming during reverse transcription and sequencing-based readout of reverse transcription termination sites, making the probing of long RNAs without normalization to a no-reagent control possible. We believe that the SHAPES strategy is a useful addition to current methods for RNA structure probing and will facilitate future in vivo RNA structure probing-based on SHAPE chemistry.

## MATERIALS AND METHODS

### Hydrolysis rate measurements

*N*-propanone isatoic anhydride (NPIA) was obtained from Enamine Ltd., product number EN300-78111 and *N*-methyl isatoic anhydride (NMIA) was from Life Technologies, product number M-25. The excitation/emission profile of NPIA and NMIA was determined using the Fluorescence Profiler feature on a NanoDrop 3300 (Thermo Scientific), having excitation optimum at 375 nm and emission optimum at 440 nm. To determine hydrolysis reaction rates, 1 μL 10 mM NPIA or NMIA was added to 1 mL reaction buffer (100 mM potassium phosphate pH 8.0, 10% v/v DMSO, 250 mM NaCl), and the formation of the hydrolysis product was measured at 25°C for 50 min with 30-sec time intervals as an increase in fluorescence. Nonlinear regression (exponential rise to maximum, single, three parameters) was fitted to the data using the SigmaPlot v11.0 software and the equation *f* = *y*_0_ + *a* × [1 − exp(−*b* × *x*)], where *y*_0_ is the offset, *a* is the amplitude, and *b* is the decay constant. We found that NMIA had a *b* value of 0.042 and a half-life of 13.5 min. NPIA had a *b* value of 0.088 and a half-life of 5.9 min.

### ATP gel shift

Radiolabeled dCTP and ATP (50,000 counts per min [cpm]/µL) was incubated with 50 mM NPIA or DMSO in 100 mM HEPES pH 8.0, 6 mM MgCl_2_, and 100 mM NaCl (1 h, 37°C, total volume 10 µL). One microliter of each of the reactions was mixed with 2 µL 1 M Na-citrate pH 6.0 and 6.75 µL 50 mM biotin (long arm) hydrazide dissolved in DMSO, and water to a final volume of 28.75 µL. A control reagent with DMSO instead of biotin (long arm) hydrazide was included. The reactions were incubated 12 h at 25°C in the dark. One microliter of each reaction was mixed with 9 µL water and 2 µL 6× loading buffer, and loaded on a 30% native polyacrylamide gel (29:1 Acrylamide/Bis-acrylamide, 1% TBE). After electrophoresis (14 W, 1 h), the result was analyzed with phosphorimaging (STORM, Molecular Dynamics).

### RNase P specificity domain RNA preparation

A DNA template containing the sequence encoding the *B. subtilis* RNase P specificity domain inserted in a structure cassette as previously described (Merino et al. 2005; Lucks et al. 2011) was synthesized de novo (MWG Eurofins Operon). The DNA sequence was inserted into the standard vector pEX-A, and the plasmid was transformed into One Shot TOP10 chemically competent *E. coli* cells (Invitrogen). The RNase P specificity domain sequence was verified by Sanger sequencing. The plasmid was linearized with BsaI-HF (New England Biolabs) and used as a template for in vitro transcription. The in vitro transcription reaction (200 μL, 37°C, 4 h) contained T7 RNA polymerase, 2 mM of each NTP, 40 mM Tris–HCl pH 8.0, 6 mM MgCl_2_, 1 mM Spermidin, 5 mM DTT, and 1 μg linearized DNA template. After transcription, the RNA was ethanol precipitated, centrifuged and resolved on a 5% polyacrylamide, 7 M urea, 1× TBE gel. It was detected with UV shadowing as a single band on the gel. A gel slice containing the band was cut out, and the RNA was eluted overnight in a buffer containing phenol, 250 mM NaOAc and 1 mM EDTA. The aqueous phase was extracted with chloroform, and after ethanol precipitation and centrifugation, the RNA was resuspended in water. The RNA was folded as previously described (Kjems et al. 1998) with modifications. Briefly, RNA (1.5 μg) was heated in water to 95°C for 2 min, and placed on ice for 1 min. Folding buffer was added to a concentration of 100 mM HEPES pH 8.0 and 100 mM NaCl, and the RNA solution was incubated at 37°C for 10 min. After addition of MgCl_2_ to10 mM the RNA was incubated for an additional 10 min at 37°C.

### Total RNA preparation

Total RNA was isolated from the *E. coli* strain MRE600 (a gift from Birte Vester, University of Southern Denmark), under nondenaturing conditions as previously described (Deigan et al. 2009). Bacteria were grown in 1.5 mL LB medium to mid-log phase (OD_600_∼0.6), and the cells were transferred to 4°C for 20 min. After collection by centrifugation (5 min, 4°C, 17,000*g*), the cells were resuspended in 1 mL buffer A (15 mM Tris–HCl (pH 8), 450 mM sucrose, and 8 mM EDTA), and lysozyme (Sigma-Aldrich) was added to a final concentration of 0.4 mg/mL. The cells were incubated at 22°C for 5 min and kept on ice for 10 min. The protoplasts were collected by centrifugation (5 min, 4°C, 5000*g*), and resuspended in 120 µL buffer B (50 mM HEPES [pH 8.0], 200 mM NaCl, 5 mM MgCl_2_, and 1.5% [wt/vol] SDS). After 5-min incubation at 22°C and 5 min on ice, 30 µL buffer C (50 mM HEPES [pH 8.0], 1 M potassium acetate, and 5 mM MgCl_2_) was added, and the sample was centrifuged (5 min, 4°C, 17,000*g*) to precipitate the SDS. The pelleted SDS was discarded and the buffer was exchanged by gel filtration using a NucAway column (Ambion) that was preequilibrated with buffer D (50 mM HEPES [pH 8.0], 200 mM potassium acetate [pH 8.0], 5 mM MgCl_2_). The RNA was then extracted three times with 1 vol. phenol (pH 8.0):chloroform:isoamyl alcohol; 25:24:1, and three times with chloroform. The RNA quality was verified with a Bioanalyzer Pico assay (Agilent) before structure probing.

### SHAPE structure probing

Folded RNase P or total RNA from *E. coli* was treated (37°C, 45 min) with 1/10 vol. NPIA or NMIA dissolved in DMSO (60 mM), or treated with DMSO as a control. After probing, the RNA was precipitated with ethanol, centrifuged and the pelleted RNA was dissolved in water.

### Primer extension

A primer designed to match the 3′ structure cassette of the RNase P construct was used for reverse transcription of RNase P (2.5 μL of 100 μM DNA primer RT_structure_cassette, primer sequence listed in Supplemental Table 1). Reverse transcription of total RNA was carried out with random priming (1 µL of 50 μM DNA primer, RT_random_primer, primer sequence listed in Supplemental Table 1). Reverse transcription was performed as described previously (Takahashi et al. 2012) with modifications. Annealing reactions had a total volume of 7 µL, and were carried out at 65°C for 5 min, followed by incubation at 37°C for 1 min. The solution was placed on ice, and 30 μL enzyme mix (7.5 μL 5× Reverse transcription buffer [250 mM HEPES pH 8.3, 375 mM KCl, 15 mM MgCl_2_], 7.5 μL 2.5 mM dNTPs, 7.5 μL 3.3 M/0.6 M sorbitol/trehalose mix, 2.5 μL PrimeScript Reverse Transcriptase (TAKARA), and 5 μL water) was added. After mixing, the reaction was incubated 1 min at 25°C, 30 min at 42°C, 10 min at 50°C, 10 min at 56°C, and 10 min at 60°C, and kept on ice before purification. The reaction conditions for RNase P and total RNA primer extension were the same, except that the first step in the reverse transcription (1 min at 25°C) was omitted for RNase P. The cDNA/RNA hybrids were purified using Agencourt RNAClean XP kit (cDNA/RNA:beads ratio 1:1.8), as previously described (Takahashi et al. 2012) The cDNA/RNA hybrids were eluted in 40 µL water.

### Biotinylation and selection of full-length cDNA

Biotinylation, RNase I treatment and full-length cDNA selection were performed as previously described (Takahashi et al. 2012) with modifications. In brief, 4 μL of 1 M Na-citrate (pH 6.0), and 13.5 μL 15 mM biotin (long arm) hydrazide (Vector Labs) in DMSO were added to the 40 μL cDNA/RNA sample. The reaction was incubated at 23°C for 15 h in the dark. After biotinylation, 6 μL of Tris–HCl (pH 8.5) and 1 μL of 0.5 mM EDTA (pH 5.0) was added, and the cDNA/RNA hybrids were treated with 5 µL 10 U/µL RNase I (Fermentas) at 37°C for 30 min. At the end of incubation the reaction was heated to 65°C for 5 min to denature short RNA–cDNA duplexes. The cDNA/RNA hybrids were then recovered by ethanol precipitation and centrifugation, and resuspended in 40 μL water. For each reaction, 100 μL MPG Streptavidin (PureBiotech) beads were used. The beads were blocked with 1.5 μL 20 μg/μL *E. coli* tRNA mix for 30 min at room temperature, separated from the supernatant on a magnetic stand and washed twice with 50 μL wash buffer 1 (4.5 M NaCl, 50 mM EDTA pH 8.0) before being resuspended in 80 μL wash buffer 1. The 40 μL cDNA/RNA sample was added to the beads, and the sample was incubated 30 min at room temperature, vortexing every 5 min. The beads were separated on the magnetic stand, and the supernatant was discarded. The beads were then extensively washed (wash buffer 1 [one time], wash buffer 2 [300 mM NaCl, 1 mM EDTA pH 8.0] [one time], wash buffer 3 [20 mM Tris–HCl pH 8.5, 1 mM EDTA pH 8.0, 500 mM NaOAc pH 6.1, 0.4% SDS] [two times], wash buffer 4 [10 mM Tris–HCl pH 8.5, 1 mM EDTA pH 8.0, 500 mM NaOAc pH 6.1] [two times]), using 150 μL of the buffers in each wash. To elute the full-length cDNA, 60 μL 50 mM NaOH were added to the beads, and they were incubated 10 min at room temperature. After separation on the magnetic stand, the supernatant was recovered and kept on ice. To neutralize the solution, 12 μL of 1 M Tris–HCl (pH 7) was added. The cDNA was then precipitated with ethanol, centrifuged, and resuspended in 8 μL water.

### Library preparation

cDNA was diluted to the concentration 0.66 ng/μL (RNase P) or 0.5 ng/µL (*E. coli* total RNA). For ligation, 3 µL cDNA was mixed with 7 μL ligation mix (prepared by mixing 1 volume Circligase 10× reaction buffer, 0.5 volume 1 mM ATP and 50 mM MnCl_2_, 2 volume 50% PEG 6000 and 5 M betaine, 1 volume 100 µM Ligation_adapter oligonucleotide and 0.5 volume Circligase [Epicentre]). The mixture was incubated for 2 h at 60°C, then 1 h at 68°C and 10 min at 80°C. After the cDNA was precipitated with ethanol, it was dissolved in 30 μL H_2_O (RNase P) or 10 µL H_2_O (*E. coli* total RNA). PCR was performed using 5 μL of the adapter-ligated cDNA, mixed with 45 μL PCR mix (prepared as a mixture of three volumes of PCR_forward primer, 2.5 volumes of PCR_reverse_index.# (different barcode for each reaction) reverse primer (Supplemental Table 1), 10 volumes of Phusion 5× HF buffer, 4 volumes of 2.5 mM dNTPs, 27.5 volume H_2_O, and 1 volume Phusion Polymerase). Reactions underwent thermal cycling with the following program: 1× (98°C for 3 min), 4× (98°C for 80 sec; 64°C for 15 sec; 72°C for 30 sec), 15× (98°C for 80 sec; 72°C for 45 sec), 1× (72°C for 5 min). The generated PCR amplicons were purified with Ampure XP beads using the ratio 1:1.8 as previously described (Takahashi et al. 2012) and eluted in 20 μL 10 mM Tris–HCl pH 8.3. The purified samples were analyzed with a Bioanalyzer DNA 1000 assay (Agilent) to estimate the concentrations, pooled and size-selected (200–600 bp range) on an E-gel 2% SizeSelect gel (Invitrogen), which were further concentrated with a PCR purification column (Qiagen). The RNase P library was sequenced on the Illumina HiSeq system with the 1 × 50 protocol, whereas the total *E. coli* RNA library was sequenced with the 100-nt paired-end protocol. The raw sequencing data are available here: http://people.binf.ku.dk/~jvinther/data/SHAPES-Seq/.

### Reads preprocessing and mapping

Reads from the *E. coli* ribosome probing experiment were processed with the Cutadapt (Martin 2012) utility to remove remaining adapter sequence. The first read from each pair was processed with options “-a AGATCGGAAGAGCACACGTCT -q 17” and the second with “-a AGATCGGAAGAGCGTCGTGTAGGGAAAGAGTGT -q 17”. After adapter trimming the pairs containing a read shorter than 15 nt were discarded. The reads were mapped to the reference index composed of (a) a Trinity (Grabherr et al. 2011) assembly of the 16S ribosomal RNA of the MRE600 strain (based on a previous experiment, the differences from the chain A in PDB:3OFA were r.80a > c, r.89u > g, r.93u > c and r.1498u > g), and (b) sequences of the spiked-in β-actin and RNase P fragments. The mapping was performed with the Bowtie 2.1.0 program (Langmead and Salzberg 2012) using options “-N 1 -L 15 --norc -X 700”. Following mapping, untemplated nucleotides within the first 3 positions that potentially could have been added by the terminal transferase activity of the reverse transcriptase were trimmed as previously described (Kielpinski et al. 2013).

Mapping of reads for the *B. subtilis* RNase P experiment was performed with the Bowtie program (Langmead et al. 2009) using default options followed by trimming of the untemplated nucleotides (Kielpinski et al. 2013).

### Data analysis

Data analysis was carried out in R (R-Core-Team 2014). For both the *RNase P* specificity domain RNA and the 16S rRNA, the termination counts were obtained for each position by summing counts for all fragments having a 5′ end that terminated at the position immediately before the position in question. Correlation between the RNase P data sets was calculated using the Spearman's rank correlation coefficient, *R*. For the 16S rRNA, SHAPE reactivities were calculated by sliding a 70-nt window across the sequence and in each window removing outliers by 90% Winsorization (all values above the 95th percentile are set to the 95th percentile), followed by normalization with the 95th percentile to give reactivities between 0 and 1. The final reactivity for each position was obtained as the average of the reactivities obtained for that particular position in the different windows.

Secondary structure annotation for the *B. subtilis* RNase P specificity domain RNA and the *E. coli* 16S rRNA was obtained from the RNA Mapping Database (Cordero et al. 2012) and CRW database (Cannone et al. 2002), respectively. The secondary structures and the corresponding SHAPES values were visualized using the VARNA Java applet (Darty et al. 2009). The pROC R package (Robin et al. 2011) was used to calculate the structure signal (AUC) present in the different data sets using the secondary structure annotations as the binary classifier. For ROC curve analysis, positions 83–244 of the RNase P RNA and 1–1350 of the 16S RNA was used and for the 16S rRNA, only positions having a ribose accessibility >3 Å^2^ was used in the analysis. The 16S rRNA accessibility values were calculated as previously described (Kielpinski and Vinther 2014). The capillary SHAPE data for the *E. coli* 16S rRNA (Deigan et al. 2009) were obtained from the Weeks Laboratory home page (http://www.chem.unc.edu/rna/data-files/Deigan_DATA.zip) and the RNase P RNA data (Mortimer and Weeks 2007) were provided by Kevin Weeks. The SHAPE-Seq data (Lucks et al. 2011) were obtained from the RMDB database (Cordero et al. 2012) (http://rmdb.stanford.edu/repository/detail/RNASEP_SHP_0000). For normalization of the RNase P and 16S rRNA data with the DMSO control, the coverage at each position was calculated by summing the counts for all fragments spanning the position or terminating at the position immediately before the position in question. To avoid bias from size selection, fragments were only used for calculation of coverage for a given position, if the distance between the position and the priming position was at least 100 nt. A termination coverage Ratio (TCR) was calculated by dividing the termination count with the coverage for each position. For calculation of normalized values ΔTCR, we used the formula described by Weeks and colleagues (Karabiber et al. 2013):

$$\Delta \text{TCR}=\frac{{\text{TCR}}_{\text{Sample}}-\alpha \times {\text{TCR}}_{\text{Control}}}{1-\alpha \times {\text{TCR}}_{\text{Control}}}.$$

Instead of estimating the scaling factor α, we tested a wide range of α values and used the value that gave the best possible structure signal as estimated by the AUC of the ROC curve using the *E. coli* 16S rRNA secondary structure as the binary classifier.

## SUPPLEMENTAL MATERIAL

Supplemental material is available for this article.

## Supplementary Material

Supplemental Material
