# Supplementary material for: SHAPE Selection (SHAPES) enrich for RNA structure signal in SHAPE sequencing-based probing data
Source: RNA. 2015 May;21(5):1042–52. doi: 10.1261/rna.047068.114 (PMC4408784; doi:10.1261/rna.047068.114)
Supplement: Supplemental Material [file supp_21_5_1042__index.html]

SHAPE Selection (SHAPES) enrich for RNA structure signal in SHAPE sequencing-based probing data — SHAPE Selection (SHAPES) enrich for RNA structure signal in SHAPE sequencing-based probing data — Supplemental Material 

# SHAPE Selection (SHAPES) enrich for RNA structure signal in SHAPE sequencing-based probing data

## Supplemental Material

**Files in this Data Supplement:**

- Supp Material.pdf
- Supp Data Table.xlsx
