# Supplementary material for: SHAPE Selection (SHAPES) enrich for RNA structure signal in SHAPE sequencing-based probing data
Source: RNA. 2015 May;21(5):1042–52. doi: 10.1261/rna.047068.114 (PMC4408784; doi:10.1261/rna.047068.114)
Supplement: Supplemental Material [file supp_047068.114_SuppMaterial.pdf]

Supplementary information accompanying the paper:

SHAPE Selection (SHAPES) enrich for RNA structure signal in SHAPE sequencing based probing data

Line Dahl Poulsen[1], Lukasz Jan Kielpinski[1], Sofie Salama[2], Anders Krogh[1], Jeppe Vinther[1,]*,

[1]Department of Biology, University of Copenhagen, Ole Maaløes Vej 5, DK-2200 Copenhagen N, Denmark
[2]Center for Biomolecular Science and Engineering, and Howard Hughes Medical Institute, University of California Santa Cruz, Santa Cruz, California 95064, United States of America

CONTENT

Supplementary figure 1

Supplementary table 1

Supplementary Figure 1. SHAPES reactivity values from the NPIA selected sample shown on the E. coli 16S rRNA secondary structure. White positions corresponds to no data.

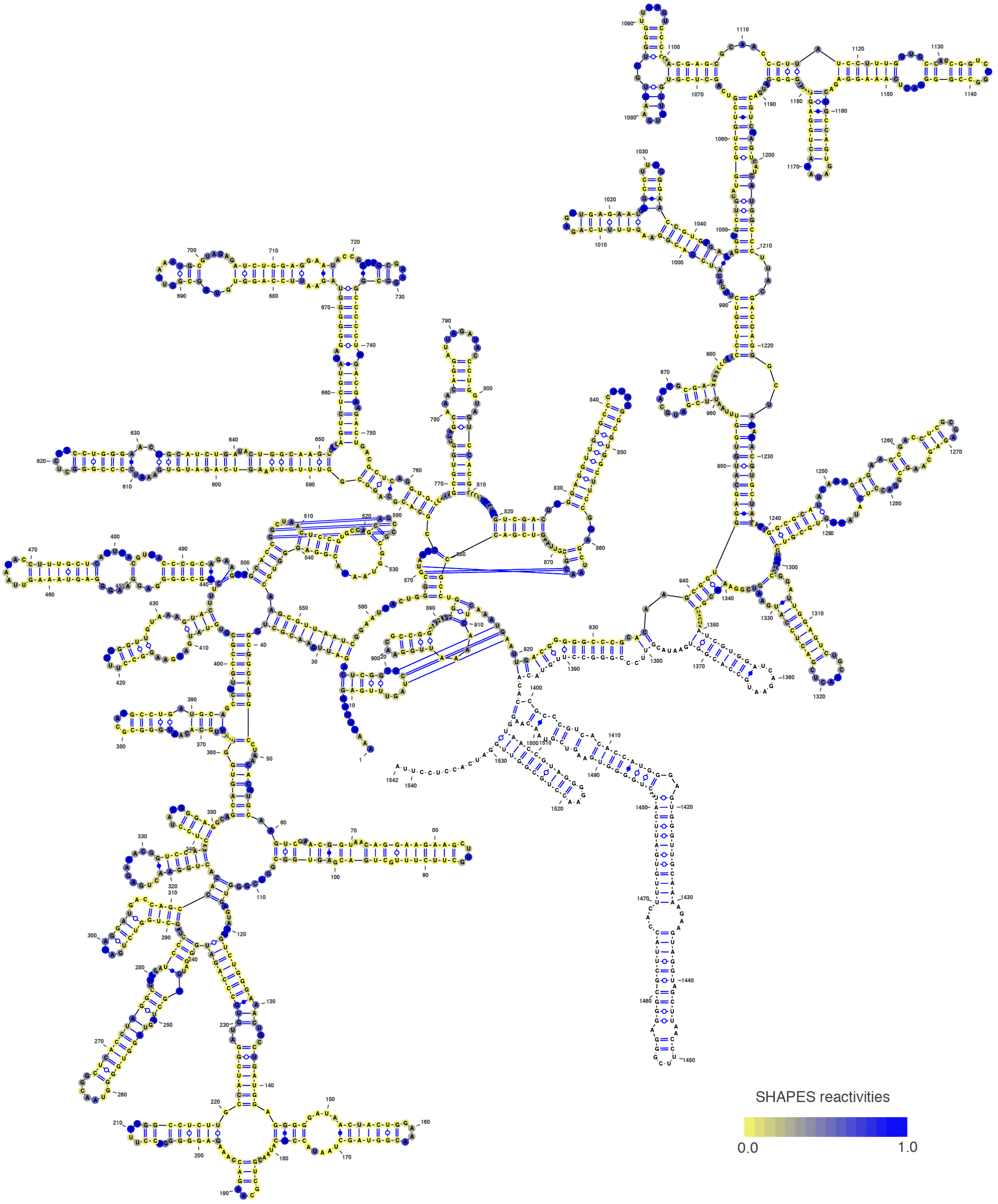

Supplementary Table 1. Oligonucleotides used in the study

**Oligonucleotide name**	Oligonucleotide sequence (5-' to 3'-direction)
**RT_structure_cassette**	AGACGTGTGCTCTTCCGATCTGAACCGGACCGAAGCCCG
**RT_random_primer**	AGACGTGTGCTCTTCCGATCTNNNNNNNNS
**Ligation_adapter**	PHO-AGATCGGAAGAGCGTCGTGTAGGGAAAGAGTGT-3NHC3
**PCR_forward**	AATGATACGGCGACCACCGAGATCTACACTCTTTCCCTACACGACGCT
**PCR_reverse_index.1**	CAAGCAGAAGACGGCATACGAGATCGTGATGTGACTGGAGTTCAGACGTGT GCTCTTCCGATCT
**PCR_reverse_index.2**	CAAGCAGAAGACGGCATACGAGATACATCGGTGACTGGAGTTCAGACGTGT GCTCTTCCGATCT
**PCR_reverse_index.5**	CAAGCAGAAGACGGCATACGAGATCACTGTGTGACTGGAGTTCAGACGTGT GCTCTTCCGATCT
**PCR_reverse_index.6**	CAAGCAGAAGACGGCATACGAGATATTGGCGTGACTGGAGTTCAGACGTGT GCTCTTCCGATCT
**PCR_reverse_index.11**	CAAGCAGAAGACGGCATACGAGATGTAGCCGTGACTGGAGTTCAGACGTGT GCTCTTCCGATCT
**PCR_reverse_index.12**	CAAGCAGAAGACGGCATACGAGATTACAAGGTGACTGGAGTTCAGACGTGT GCTCTTCCGATCT
**PCR_reverse_index.14**	CAAGCAGAAGACGGCATACGAGATGGAACTGTGACTGGAGTTCAGACGTGT GCTCTTCCGATCT
**PCR_reverse_index.15**	CAAGCAGAAGACGGCATACGAGATTGACATGTGACTGGAGTTCAGACGTGT GCTCTTCCGATCT

Oligonucleotide sequences © 2007-2009 Illumina, Inc. All rights reserved.
